# Learning depends on the information conveyed by temporal relationships between events and is reflected in the dopamine response to cues

**DOI:** 10.1126/sciadv.adi7137

**Published:** 2024-09-06

**Authors:** Peter D. Balsam, Eleanor H. Simpson, Kathleen Taylor, Abigail Kalmbach, Charles R. Gallistel

**Affiliations:** ^1^ Department of Psychology, Barnard College, New York City, NY, USA.; ^2^ Columbia University and New York State Psychiatric Institute, New York City, NY, USA.; ^3^ Department of Psychology and the Rutgers Center for Cognitive Science, Rutgers University, New Brunswick, NJ, USA.

## Abstract

Contemporary theories guiding the search for neural mechanisms of learning and memory assume that associative learning results from the temporal pairing of cues and reinforcers resulting in coincident activation of associated neurons, strengthening their synaptic connection. While enduring, this framework has limitations: Temporal pairing–based models of learning do not fit with many experimental observations and cannot be used to make quantitative predictions about behavior. Here, we present behavioral data that support an alternative, information-theoretic conception: The amount of information that cues provide about the timing of reward delivery predicts behavior. Furthermore, this approach accounts for the rate and depth of both inhibitory and excitatory learning across paradigms and species. We also show that dopamine release in the ventral striatum reflects cue-predicted changes in reinforcement rates consistent with subjects understanding temporal relationships between task events. Our results reshape the conceptual and biological framework for understanding associative learning.

## Teaser

The amount of information that cues provide about the timing of reward availability determines the rate and strength of learning.

## INTRODUCTION

To understand associative learning, there are four questions that need to be answered. (i) What are the conditions that give rise to the learning? (ii) What are the contents of the learning? (iii) How does that knowledge relate to behavior? (iv) What are the neural mechanisms that underlie the answers to the first three questions? The broadly accepted answer to the first question is the idea that two events become associated because of their close temporal proximity. Models of associative learning based on temporal pairing (*1*–*4*) motivated subsequent trial-based and state-action–based computational models of reinforcement learning (*5*–*9*). Trial- or state-based models have been developed to account for a wide range of phenomena and provide a productive framework for answering these questions. This approach has generated a large number of empirical studies that show a conditioned stimulus (CS) conveys information about many aspects of a reinforcer [see (*10*)], including the type and amount of reinforcement received (*10*), the trial structure during partial reinforcement (*11*–*13*), the rate of reinforcement (*14*–*16*), and the temporal relationships between stimuli (*17*, *18*). Furthermore, pairing-based models have been generalized to deal with the associations formed between responses and outcomes (*19*) and have even been used to model which anticipatory responses will reflect the underlying learning (*3*). The third question is yet to be fully addressed (*20*, *21*). Because there are no general principles describing how to map theorized associative strength into a precise strength of behavior, tests of these models must rely on ordinal predictions about behavior. However, the brain mechanisms that underlie behavior map the underlying learning into quantitative variation in behavior.

Here, we present behavioral data that support an information-theoretic conception of associative learning that resolves the first and third questions posed above. Temporal information that cues provide about the timing of reward delivery determines both the rate of learning and a quantitative relation between this knowledge and behavior. Furthermore, we show that this approach accounts for the rate and depth of both inhibitory and excitatory learning across paradigms and species. We also show that dopamine release in the ventral striatum reflects cue-predicted changes in reinforcement rates consistent with subjects understanding temporal relationships between task events.

Our approach was inspired by evidence that temporal pairing is neither necessary nor sufficient for the emergence of conditioned responding (*22*–*29*). Some of the well-established learning phenomena that cannot be explained by temporal pairing as the core mechanism of learning include the following: (i) Intermixing unreinforced stimulus presentations with reinforced presentations (partial reinforcement) does not slow learning and can make performance more robust to extinction (*30*–*35*). (ii) Increasing the number of reinforced trials per unit time has no effect on the training time required to obtain a conditioned response (*36*). (iii) Learning can occur despite long delays (minutes or even hours) between cues and reinforcers in what is called trace conditioning (*37*–*39*). (iv) Knowledge about temporal relationships between cues and reinforcements can be transferred across stimuli and response types (*40*, *41*).

**Fig. 1.**
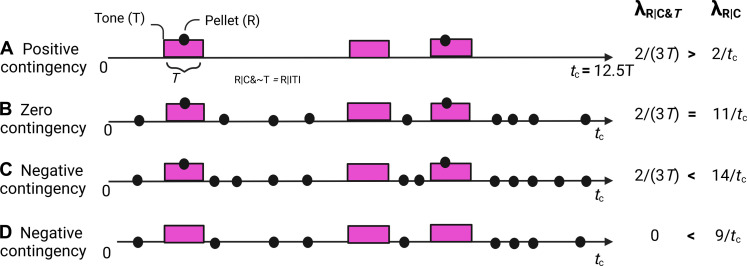
Contingency in conditioning. The onset of the Tone may signal an increase in the rate of reinforcement compared to the contextual rate calculated over the entire session time, *t*_c_ (**A**); have no effect on it (**B**); or signal a decrease in it (**C** and **D**). In our experiments, the dots (reinforcers, R) are food pellets and predictive events are Tones (denoted by T) with a duration denoted by *T*. λ_R∣C_ *=* the contextual rate of reinforcement (the number of reinforcers obtained in the chamber, *N*_R_ / amount of time in the chamber, *t*_c_). λ_R∣C&T_ *=* rate of reinforcement during the Tone, T, in the context of the test chamber, C. λ_R∣C~T_ = rate of reinforcement in the context alone without T (reinforcement rate during the ITI, can also be denoted λ_R∣ITI_).

To explain many of these results using a temporal-pairing framework, theorists posit that cues and intertrial intervals (ITIs) are composed of multiple “trials” that are either reinforced or nonreinforced, thus creating a hidden or hypothetical trial structure (*1*, *2*, *5*). The analysis of the contingency manipulations depicted in Fig. 1 helps illustrate this approach. The probability of reinforcement during the cue (a Tone, denoted by T) is identical in protocols in Fig. 1 (A to C); therefore, simple temporal pairing–based theories predict identical behavioral outcomes. Instead, protocol in Fig. 1A produces more responding during the Tone than during the ITI, protocol in Fig. 1B produces no differential responding, and protocol in Fig. 1C results in less responding during the Tone than the ITI. Using a temporal-pairing model to account for this result requires imposing a hypothetical trial structure and then claiming that the different outcomes from protocols in Fig. 1 (A to C) result from the contrast between the numbers of reinforced and nonreinforced trials that occur in the presence of a cue compared to an ITI. The problem with this explanation is that there is no principled way to specify when trials occur nor how many there are (*42*, *43*). In protocols in Fig. 1 (A to C), reinforcements are scheduled at random times during T, thus, reinforcement is equally probable at every moment within T. Because there are no specifiable times at which nonreinforcement may be deemed to have occurred, nonreinforced trials are uncountable. Also, to explain the temporally patterned learned anticipatory behavior that occurs within Ts of fixed duration, the duration of the assumed trials must be briefer than the duration of T. As the hypothetical trials are made briefer and more numerous, the probability of reinforcement becomes smaller and smaller; in the limit, it vanishes. We are unaware of any associative trial-, state-, or action-based models of learning that do not depend on the use of hypothetical or “hidden” trials.

In addition to this problematic explanation of the behavioral observations in the experiments depicted in Fig. 1, another limitation of all trial-, state-, and action-based models that derive from the original Rescorla-Wagner model is that they do not predict behavior; rather, they predict the hypothetical construct of associative strength. The unspecified link between associative strength and behavior allows only for qualitative, ordinal predictions of behavior not for the quantitative predictions ultimately needed to understand brain computations.

The alternate approach that we take strips learning down to the essential element of adaptive behavior—performance. This approach does not investigate the role of important behavioral factors such as motivational drive, memory contents, sensory processing, etc. Instead, by considering that only measurable information cues convey about the timing of events, many quantitative aspects of conditioned responding are explained by simple and explicit closed-form equations that directly predict behavioral results (*42*, *43*). This information-theoretic conception of learning focuses selectively on the temporal content of experience and rests on extensive experimental evidence that subjects: (i) measure and store in memory the durations of the intervals between observable changes in state, such as cue onsets and offsets and placements into and removals from test chambers; (ii) count meaningful events such as reinforcements, which occur during these intervals; and (iii) use this information to compute the rates at which events occur. Unlike using hypothetical trials to compute probability of reinforcement and nonreinforcement, interval durations, event counts, and rates are easily measured quantitative aspects of a subject’s experience. They constitute a protocol’s metric temporal structure.

In protocols in Fig. 1 (A to D), because reinforcers (Rs) are randomly distributed in time within a given state (tone on or off), there are no specific times at which reward can be expected, but the animal has objective information about the rate of reinforcement in each state: the state of being in the test chamber (denoted by C), which comprises two possibilities: the state of being in the chamber in the presence of the Tone (C&T) and the state of being in the chamber in the absence of the Tone (C&~T). The rate of reinforcement during the Tone (λ_R∣C&T_) relative to the overall rate of reinforcers for the session (λ_R∣C_) determines whether the contingency between Tone and reinforcers is positive (Fig. 1A), zero (Fig. 1B), or negative (Fig. 1C and D).

In protocol 1d, there is a perfect negative contingency between T and reinforcer (R) and subjects readily learn the duration of the Tone and its meaning. The complete absence of temporal pairing between T and R makes this rarely used protocol centrally important to understand learning. Here, we show that in protocols with perfect negative contingency, asymptotic performance, the rate of learning, and the pattern of responding within T depend on temporal structure in the same way they do for positively contingent protocols. We also identify a real-time neural correlate of reward state: ventral striatal dopamine.

## RESULTS

### Negatively contingent cues do not inhibit responding

**Fig. 2.**
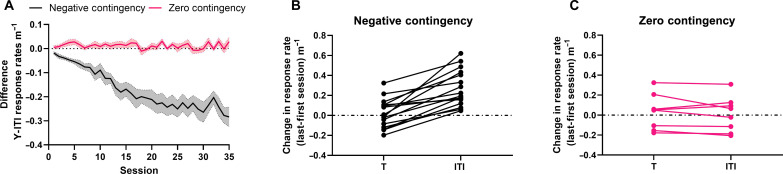
Change in responding over training. (**A**) Differences in the T-ITI response rates as a function of session number. Negative-contingency protocol: Rats were rewarded on average every 20 s during 20-s-long ITIs but not during 40-s-long Tones. Zero-contingency protocol: Rewards and Tones were random. Solid line = mean, shading = SEM. (**B**) Individual subject’s change in response rates between the first and last session of training under the negative-contingency protocol. Change scores for each subject are plotted separately for T and ITI. (**C**) Changes under the zero-contingency protocol. Negative *N* = 16, Zero *N* = 8.

Here, we show in Pavlovian protocols as in instrumental ones [e.g., (*44*)] that rodents can learn the meaning of cues that predict when rewards are unavailable (see protocol in Fig. 1D and Fig. 2A). Temporal pairing–based theories that posit that nonreinforced trials decrease associative strength and/or decrease the value of a state, predict that the difference between Tone and ITI response rates increase because subjects reduce responding during the nonreinforced Tones. On the contrary, the average change in Tone response rates was close to 0. The difference between Tone and ITI (Tone-ITI) response rate grew larger from the first to last session (Fig. 2A) because every subject increased their response rate during the ITIs and for every subject, this increase was greater than any change in response rates during the Tones (Fig. 2B). In contrast, in the zero-contingency protocol (protocol Fig. 1B) changes in response rates from the first to last session (increase or decrease) are the same for both T and ITI (Fig. 2C). This result is explained by a model in which the magnitude of the difference in response rates depends on the Shannon information conveyed by Tone offsets about the expected increment in the rate of reinforcement, rather than on the probabilities of reinforcement and nonreinforcement in the two stimulus conditions.

A predictive event (e.g., a CS) provides a subject with information about an impending reinforcement when it reduces the subject’s uncertainty about the wait for it. When a rodent is placed in a test chamber where food-pellet reinforcements drop into a hopper at random intervals (RIs), there is uncertainty about when to expect each pellet. In information theoretic terms, the information available about the wait for reinforcement is measured by the differential entropy of an exponential distribution of waits for reinforcement (*45*). The differential entropy is a function only of the rate parameter of this distribution (λ_R∣C_, in Fig. 1), which is one over the average wait. The sound of a pellet dropping into the hopper reduces a subject’s uncertainty about the wait to 0, thereby conveying all of the available information.

An informative CS partitions its context into higher and lower rate intervals. In positively contingent protocols (e.g., Fig. 1A), a Tone onset signals an increase in the rate, λ_R_, at which reinforcement occurs (a reduction in the average wait). In a negatively contingent protocol, Tone offset signals an increase in the rate of reinforcement. In either case, the greater the increase in reinforcement rate, the greater the reduction in uncertainty about the wait until reinforcement. The amount of information that the CS onset or offset conveys depends on a ratio between the rate during the higher-rate portions—λ_R∣C&CS_ or λ_R∣C&~CS_—and the contextual reinforcement rate, λ_R∣C_, which is the cumulative number of reinforcers delivered divided by the cumulative time in the experimental chamber (*14*). We call this unitless ratio informativeness (*14*) and denote it by a lower-case iota, ɩ. When Tone onset signals the increase, ι = λ_R∣C&CS_/λ_R∣C_; when Tone offset signals the increase, ι = λ_R∣C&~CS_/λ_R∣C._

In negatively contingent protocols (e.g., Fig. 1, C and D), Tone offsets predict an increase in the rate of reinforcement thus driving the increased rate of responding observed during the ITIs (Fig. 2B). The informativeness of a Tone offset is the ratio between the rate of reinforcement expected in the absence of T, λ_R∣C&~T_, and the contextual rate of reinforcement, λ_R∣C_. This information driven view of learning implies that inhibitory conditioning involves the same learning processes as excitatory conditioning; the difference lies only in whether it is the Tone onset or the Tone offset that is informative about the increase in λ_R_.

### Increasing the number of ITI-reinforcement pairings can slow learning in a negative contingency

Temporal pairing–based theories of learning suppose that associative strength increases with the number of reinforcers. Therefore, these theories predict that in negatively contingent protocols, increasing ITI durations (which increases the expected number of pellets during an ITI and the probability that an ITI will be reinforced at least once) would speed learning and enhance performance. By the same logic, such theories predict that increasing Tone duration (which does not alter the fact that Tones are never reinforced nor alter the number of expected reinforcements in the ITI) might speed the learning (because of an increase in hypothetical extinction trials) but would have no effect on the asymptotic performance. To test these predictions, we trained cohorts of rats on negatively contingent protocols with Tones (T) of different durations (*T*) and ITIs of different lengths. To determine the effect of manipulating these protocol variables on learning we obtained two different rates of learning: Unconditioned Stimuli (USs; pellets) to acquisition and trials to acquisition. In both cases, acquisition was determined by calculating the strength of the evidence for a negative slope in the cumulative record of the difference in estimated poke rates (Tone poke rate - ITI poke rate, adjusted for time collecting rewards) computed using the cumulative coding cost statistic described in the methods. Acquisition values in all plots represent the US (pellet) or trial number on which the strength of the evidence for poke rate difference exceeded α = 0.01.

**Fig. 3.**
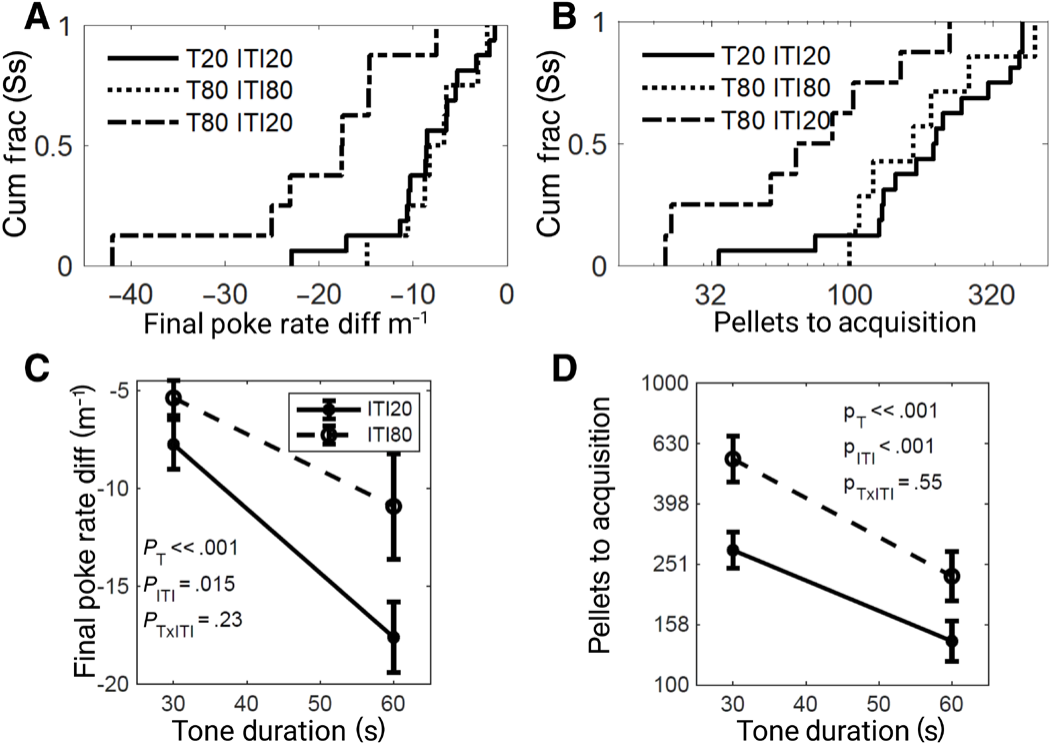
Final response rates and acquisition speed. (**A**) The cumulative distributions of asymptotic poke rate differences (Tone-ITI) for three groups of rats under the negatively contingent protocols: T20:ITI20, T80:ITI80, and T80:ITI20. (**B**) cumulative distributions of pellets to acquisition from the same three groups. (**C**) Asymptotic poke rate differences (Tone-ITI) for four groups of rats under the negatively contingent protocols: T30:ITI20, T30:ITI80, T60:ITI20, and T60:ITI80. (**D**) Pellets to acquisition from the same four groups. (Acquisition is defined as the cumulative number of pellets earned when the strength of the evidence for a negative difference in Tone poke rate - ITI poke rate had reached significance, α = 0.01.)

Cumulative distribution plots of the difference in response rates during T and ITI after acquisition of three different protocols; T20:ITI20, T80:ITI80, and T80:ITI20 show the opposite of what temporal pairing theory predicts (Fig. 3, A and B). With the constant Tone duration of 80 s, a fourfold increase in the ITI duration (substantially more reinforcements) reduces the difference in T-ITI response rates in the final session. With a constant ITI duration of 20 s, a fourfold increase in the Tone duration markedly increases T-ITI response rate difference (Fig. 3A). These changes in T and ITI durations had similarly strong effects on acquisition speed (Fig. 3B). The number of pellets to acquisition was increased by longer ITIs (more reinforcement) and increased by shorter Tones, Tones that would allow fewer hypothetical nonreinforced trials of any arbitrary duration.

Symmetrically varying T and ITI duration in an additional four groups of rats T30:ITI20, T30:ITI80, T60:ITI20, and T60:ITI80 confirmed that increasing the number of reinforcers by increasing the ITI duration worsened asymptotic performance (Fig. 3C) and slowed learning (Fig. 3D). Increasing *T* improved both learning and performance. These effects were not interdependent as there was no interaction between the effects of *T* and ITI on learning or performance.

### The Informativeness of cues conveys the metric temporal structure of protocols- the durations of the intervals and their ratios

A Tone onset or offset that predicts an increase in the rate at which pellets are delivered, λ*_R_*, changes the available information because a higher rate reduces the uncertainty about the wait for reinforcement. When experiencing a range of wait times, subjects adjust the scale of their temporal representation to the range established by experienced wait durations within the context. The number of different intervals they distinguish within that range depends on the difference in duration that is just discriminable, known as the Weber fraction for duration, *w*_d_. The smaller *w*_d_ is, the more intervals can be distinguished (*46*). Put another way, the more intervals a subject distinguishes, the smaller its implicit unit of time.

Because the differential entropy of a distribution depends on the unit of time and *w*_d_, the more intervals a subject distinguishes, the greater its uncertainty about when an event will occur. Although the differential entropy depends on the unit of time, the difference in two such entropies (those related to λ_R∣C_ and μ_R∣C&T_), which we denote by Δ*H* does not, because, when the contingency is positive

$$\begin{array}{cc} \Delta H & =\left[1-\text{ln}\left(\lambda_{R|C}k^{-1}\right)\right]-\left[1-\text{ln}\left(\lambda_{R|C\&T}k^{-1}\right)\right]=\text{ln}\frac{\lambda_{R|C\&T}}{\lambda_{R|C}}\\ & =\text{ln}\frac{\mu_{R|C}}{\mu_{R|C\&T}}=\text{ln}\left(\iota_{T}\right) \end{array}$$ (1A)

and when it is negative

$$\begin{array}{cc} \Delta H & =\left[1-\text{ln}\left(\lambda_{R|C}k^{-1}\right)\right]-\left[1-\text{ln}\left(\lambda_{R|C\&∼T}k^{-1}\right)\right]\\ & =\text{ln}\frac{\lambda_{R|C\&∼T}}{\lambda_{R|C}}=\text{ln}\frac{\mu_{R|C}}{\mu_{R|C\&∼T}}=\text{ln}\left(\iota_{∼T}\right) \end{array}$$ (1B)

The temporal unit, *k* in Eq. 1, cancels out. Informativeness, *ι*, is therefore timescale invariant. So too is its logarithm, the information-theoretic measure of the association between CS onset or offset and reinforcement (*45*).

### An information-driven understanding of learning solves the problem of credit assignment

A comprehensive theory of learning must explain how subjects assign credit for reinforcement to events, states or contexts when they occur simultaneously. Our information theoretic approach solves this problem because it is based on reinforcement rates which, unlike probabilities, are additive. Therefore, the rates to be attributed to the Tone, ${\hat{\lambda }}_{R|C\&T},$ and to the context in its absence, ${\hat{\lambda }}_{R|C\&∼T}$ , are given by

$$\left[\begin{array}{c} {\hat{\lambda }}_{R|C\&T}\\ {\hat{\lambda }}_{R|C\&∼T} \end{array}\right]=P^{-1}\left[\begin{array}{c} \lambda_{R|C}\\ \lambda_{R|C\&∼T} \end{array}\right]$$ (2)

In Eq. 2, the hatted lambdas are a rational subject’s estimates of the rates to be attributed to CS&T and to the C&~T states, and the conditional probability matrix, **P**, for a negative-contingency protocol, is

$$P=\left[\begin{array}{cc} 1 & p\left(∼T|C\right)\\ 1 & 1 \end{array}\right]=\left[\begin{array}{cc} 1 & ∼T/C\\ 1 & 1 \end{array}\right]=\left[\begin{array}{cc} 1 & 1/\iota_{∼T}\\ 1 & 1 \end{array}\right]$$ (3)

where ~*T* denotes the cumulative duration of the no-Tone intervals (the ITIs), *C* the cumulative duration of the context, and *p*(~T∣C) denotes the probability that the Tone is not on at a randomly chosen moment in the context. The elements of the input vector on the right in Eq. 2 are λ_R∣C_ = *n*_R∣C_/*C*, the cumulative reinforcement count divided by the cumulative context duration, and λ_R∣C&~T_ = *n*_R∣C&~T_/~*T*, the number of reinforcements during the no-Tone intervals divided by the their cumulative duration. Thus, temporal informativeness which determines learning rate is also the key variable in the assignment of credit.

### Asymptotic performance is a monotonic function of informativeness

**Fig. 4.**
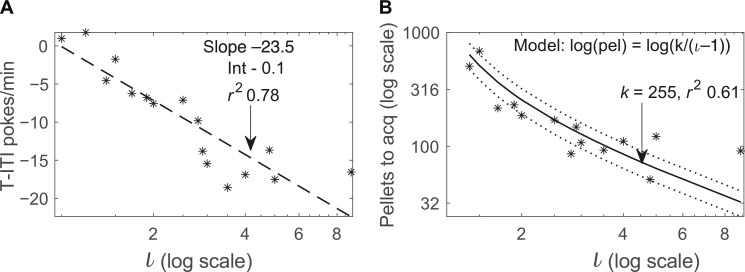
Final response rates and acquisition speed for all protocols. (**A**) The median asymptotic difference between responding during the Tones (T) and the ITI is increasingly negative as informativeness increases (scale = linear-log.) (**B**) The number of reinforcements required for the difference in the rate of responding to appear decreases in proportion to the increase in informativeness; the greater the informativeness, the faster the rate of learning. The solid line is the best-fit regression; dotted lines are confidence limits on best-fit regression (scale = log-log.). In both plots, each point represents the median value from all subjects that underwent protocols with the relevant informativeness (ɩ), for *N* see Table 1. (Acquisition is defined as the cumulative number of pellets earned when the strength of the evidence for a negative difference in Tone poke rate - ITI poke rate reached significance, α = 0.01).

To determine the explanatory power of informativeness, ι_~T_, in negatively contingent protocols, we tested different groups of rats in 14 protocols in which the informativeness of the offset of the Tone differed. We altered ι_~T_ by varying Tone durations (*T*), ~*T*, and intervals between reinforcements [inter-reward intervals (IRIs); see Table 1]. Because ι_~T_ = λ_R∣C&~T_/λ_R∣C_, increasing *T* (the duration of nonreinforced Tones) increases ι_~T_ by lowering the contextual rate of reward, λ_R∣C_. Increasing ~*T* (the ITI durations) decreases ι_~T_ by increasing λ_R∣C_.Decreasing the IRI increases the rate of reinforcement signaled by Tone offset. In these experiments, both ITIs and IRIs were drawn from exponential distributions. The Poisson processes that scheduled pellet deliveries ran only during ITIs—except in the zero-contingency (truly random) control. All Tones were of fixed duration except in special, variable *T* protocols 11 and 13. Plotting the difference in response rates during T and ~T (ITIs) at the end of training reveals that asymptotic performance is a linear function of the information conveyed by Tone offset (Fig. 4A).

**Table 1.** Protocol parameters. A total of seven experiments (six Pavlovian and one operant) were conducted with two to five groups in each experiment. Exp, Experiment ID #; Prot, Protocol #; *N*, number of subjects; CS(T), duration of the Tone conditioned stimulus; ITI, average intertrial interval duration; IRI, average inter-reward interval duration; #Ts/ses, total number of Tones in a session, Cum.C (min), cumulative time in the training context each session; Cum.ITI (min), cumulative ITI time in each session; #Rs/ses., total number of rewards per session; λRC, contextual reward rate λ_R|ITI_, reward rate during ITI; ɩ, protocol informativeness, λ_R|C_ / λ_R|ITI_. In all experiments, Tones were of fixed duration except in experiment 3 (f, fixed duration; v, variable duration). Protocols 27 and 28 (Experiment 7) were operant, and subjects earned rewards for pressing a lever at the intervals indicated. Protocols in italics and underlined (5 and 28) have zero contingency, ɩ = 1.

Exp #	Prot #	*N*	Interval durations (s)			#Ts/ses.	Cum durations/session (min)		#Rs/ses.	Reward rates/min			Prot. ɩ
			CS (*T*)	ITI	IRI		Cum. C	Cum. ITI		T	C	ITI	
**1**	1	8	10	20	20	32	16.0	10.7	32	0	2.00	3.00	**1.5**
	2	8	20	20	20	32	21.3	10.7	32	0	1.50	3.00	**2.0**
	3	8	40	20	20	32	32.0	10.7	32	0	1.00	3.00	**3.0**
	4	8	80	20	20	32	53.3	10.7	32	0	0.60	3.00	**5.0**
	*5*	*8*	*10|20|40|80*	*20*	*20*	*32*	*30.7*	*10.7*	*32*	*1.04*	*1.04*	*1.04*	***1.0***
**2**	6	8	10	20	20	32	16.0	10.7	32	0	2.00	3.00	**1.5**
	7	8	40	20	20	32	32.0	10.7	32	0	1.00	3.00	**3.0**
	8	8	10	50	50	32	32.0	26.7	32	0	1.00	1.20	**1.2**
	9	8	10	20	20	128	64.0	42.7	128	0	2.00	3.00	**1.5**
**3**	10	8	30f	20	20	32	26.7	10.7	32	0	1.20	3.00	**2.5**
	11	8	30v	20	20	32	26.7	10.7	32	0	1.20	3.00	**2.5**
	12	8	50f	20	20	32	37.3	10.7	32	0	0.86	3.00	**3.5**
	13	8	50v	20	20	32	37.3	10.7	32	0	0.86	3.00	**3.5**
**4**	14	8	20	20	20	32	21.3	10.7	32	0	1.50	3.00	**2.0**
	15	8	40	20	20	32	32.0	10.7	32	0	1.00	3.00	**3.0**
	16	7	20	20	40	32	21.3	10.7	16	0	0.75	1.50	**2.0**
	17	8	40	20	40	32	32.0	10.7	16	0	0.50	1.50	**3.0**
	18	8	80	20	40	32	53.3	10.7	16	0	0.30	1.50	**5.0**
**5**	19	12	30	20	20	32	26.7	10.7	32	0	1.20	3.00	**2.5**
	20	12	60	20	20	32	42.7	10.7	32	0	0.75	3.00	**4.0**
	21	12	30	80	20	8	14.7	10.7	32	0	2.18	3.00	**1.4**
	22	12	60	80	20	8	18.7	10.7	32	0	1.71	3.00	**1.8**
**6**	23	8	80	80	20	8	21.3	10.7	32	0	1.50	3.00	**2.0**
	24	8	80	20	20	8	13.3	2.7	8	0	0.60	3.00	**5.0**
	25	8	160	20	20	32	96.0	10.7	32	0	0.33	3.00	**9.0**
	26	8	80	10	20	64	96.0	10.7	32	0	0.33	3.00	**9.0**
7*	27	6	80	40	20	20	40.0	13.3	40	0	1.00	3.00	**3.0**
	*28*	*4*	*80*	*40*	*60*	*20*	*40.0*	*13.3*	*40*	*1.0*	*1.00*	*1.00*	***1.0***

### Learning rate is a near linear function of informativeness

Protocol parameters that explain variance in asymptotic performance do not necessarily explain acquisition. Therefore, we examined the relationship between learning rate and protocol informativeness and found the rate of learning, denoted α, is a one-parameter linear function of informativeness (Eq. 4).

$$\alpha =\frac{1}{n_{R}}=\frac{1}{k}\left(\iota -1\right)$$ (4)

where *n*_R_ is the number of reinforcements preceding the appearance of the learned difference in response rates, and *k* is the free parameter. The value for *k* given the rat inhibitory conditioning data in Fig. 4A, equals 255. Notably, this value for *k* is the same (to within the errors in measurement) given the pigeon excitatory autoshaping data compiled in (*47*); see discussion for a direct comparison.

Because *k* is much greater than 1, which makes the intercept 1/*k* close to 0, the learning rate is an approximately scalar function of informativeness when informativeness is greater than 2. Thus, when trials to acquisition—the inverse of the learning rate—are plotted against informativeness on double logarithmic coordinates, as in Fig. 4B, the slope over most of the function is approximately −1 [see also figure 4 in (*45*)]. This is the case not only for rat inhibitory conditioning and pigeon excitatory autoshaping but also for rabbit eyeblink conditioning, a protocol with aversive reinforcement (*48*) and for trace conditioning in the head-fixed mouse (*49*).

### Informativeness has more influence than reward rate on rate of learning

To determine whether the effects of informativeness on postacquisition performance depend on the pellet scheduling parameter (inter-reward interval (IRI)], μ_R∣C&~T_, we varied it from 20 to 40 s and informativeness from 2 to 5 in different groups of rats (protocols 14 to 18, in Table 1). A twofold increase in μ_R∣C&~T_ reduce the number of pellets expected during ~T intervals from 1 to 0.5, halving both the contextual and ~T rates of reinforcement. When IRI was increased twofold without changing informativeness, the postacquisition response rate (T-ITI difference) was reduced. The magnitude of this reduction was the same for protocols with different levels of informativeness [main effect of μ_R∣C&~T_ *P* = 0.015; interaction *P* = 0.99, *F*_2,73_]. The 2-fold difference in μ_R∣C&~T_: accounted for 5% of the variance, while the 2.5-fold difference in informativeness accounted for 9% [analysis of variance (ANOVA) *P* < 0.001 for this main effect].

Because increasing the IRI (doubling μ_R∣C&~T_) reduced postacquisition performance regardless of ~T informativeness (when ι_~T_ ranged from 2 to 5), we explored whether varying the IRI also affected the rate of learning. Doubling μ_R∣C&~T_ had no impact on pellets to acquisition (*P* = 0.96, *F*_1,72_, while the difference in informativeness did have [*P* < 0.02, *F*_2,72_, η^2^ = 0.1]. Because Doubling μ_R∣C&~T_ doubled the fraction of (hypothetical) ITI trials without a pellet release, this result is incompatible with trial- and state-based models of learning. In these models, ITIs are composed of discrete trials (cf 3) and doubling the duration of intervals between reinforcements during ITIs should double the number of nonreinforced trials in which associative strength is decremented, thereby decrementing net associative strength and/or decreasing the value of a state. Clearly, this was not the case.

### Temporal response patterns are timescale invariant and predicted by the metric temporal structure of protocols

**Fig. 5.**
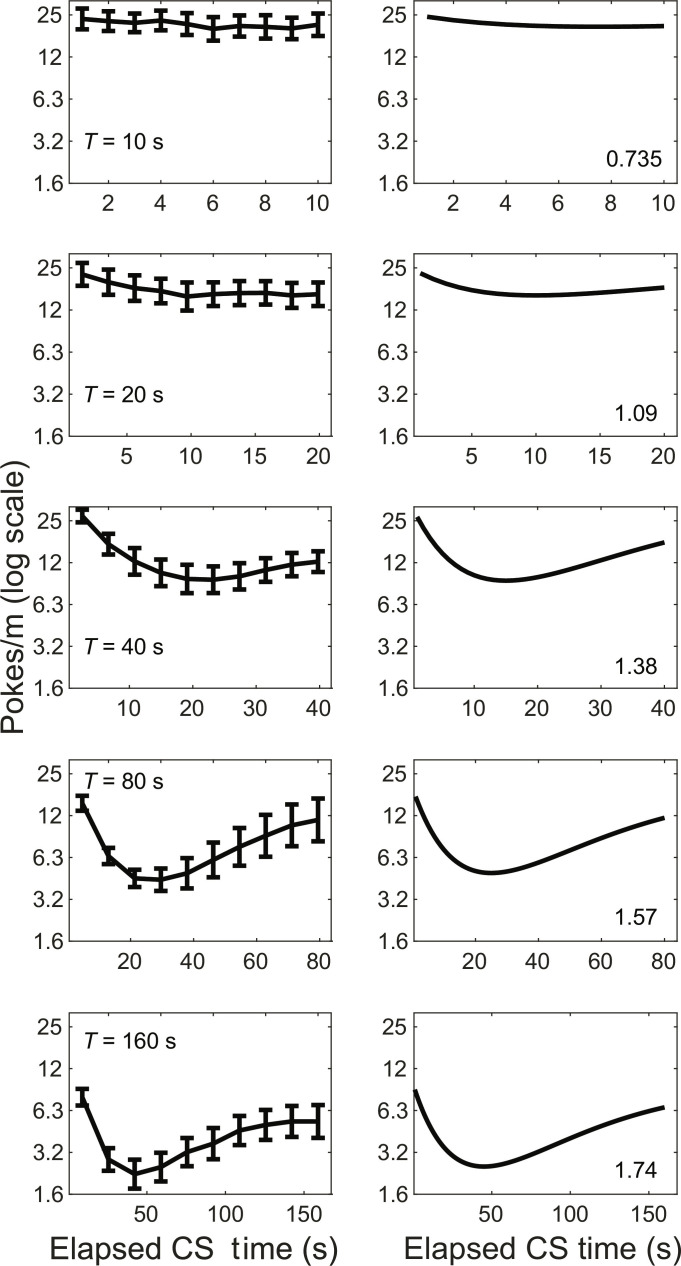
Within-CS temporal patterns of responding. (**Left column**) The profile of the response rate at successive tenths of Tone duration in the experiments with Tone lengths, *T*, given in the left panel for each row (see Table 1 for details). The average ~T interval in these examples was 20 s. The *y* axis gives the response rate (log scale) in each decile of the never-reinforced fixed-duration T intervals. (**Right column**) Results from a model in which rate-lowering and raising latencies come from exponential distributions with a time constant proportional to the mean inter-reinforcement interval in the experimental context. The number at lower right of panels on the right of each row is the number of subjective moments (just noticeable differences) elapsed at Tone offset.

The temporal pattern of responding within a fixed-duration Tone is a behavioral expression of subjects’ knowledge of the metric temporal structure of the protocol. Profiles of postacquisition responding during Tones demonstrate that subjects learned both the meaning (lack of reinforcement) and the fixed duration of the Tones. The left panels of Fig. 5 plot within-Tone response rate profiles as equivalent fractions of elapsed Tone duration for five protocols in which the average duration of the ITI was 20 s. When the fixed duration Tones are longer than 40 s (bottom three panels), average response rates decrease and then increase noticeably as more time in the Tone elapses. For shorter Tone durations (top two panels), the increase is nonexistent or minimal. The differences in the shapes of the profiles are explained by a simple model in which the temporal structure of a protocol is encoded in subjective time units. Weber’s law tells us that subjective time units scale with the expected magnitude of the waits for reinforcement. Thus, the subjective unit of time in a protocol is *w*_d_μ_R∣C_ where *w*_d_ is the Weber fraction for duration and μ_R∣C_ is the average interval between reinforcements in the experimental context.

The within-Tone patterning of responding cannot be explained by a trial-based model of associative learning or a state-based model of reinforcement learning that assumes a representation of the sequence of states but not the means and variances of their durations. Instead, three temporal metrics jointly determine whether and when subjects produce temporally patterned conditioned responding during the Tone intervals: (i) the Weber fraction, which relates subjective time units to conventional time units (seconds) in a given reinforcement context, (ii) the Tone duration in subjective time units, and (iii) the informativeness of Tone offset.

Our profile model assumes *w*_d_ = 0.16 based on a large empirical literature. We assume that the decision to decrease the response rate following Tone onset is exponentially distributed, with a time constant specified in units that scale with μ_R∣C_. The decision to resume a higher rate of responding in anticipation of Tone termination can only be made after the drop. We assume that the resumption latencies are exponentially distributed with the same time constant as the drop latencies. In that case, the probability that the response rate is at its lower value is the convolution of the exponential with itself. The convolution of an exponential with itself is the gamma distribution with shape parameter 2. On this maximally simple model, the response rate profile during the never-reinforced fixed-duration Tones is a window from *t =* 0 to *t = T* seconds on the inverted gamma probability density function—see Eq. 5

$$\lambda_{r}\left(t\right)=k\left[1-\Gamma \left(t/w_{d}\mu_{R|C},2,0.8\right)\right]$$ (5)

The first argument of the gamma distribution in Eq. 5, *t*/*w*_d_μ_R∣C_, is the time elapsed since Tone onset in subjective time units. The second argument, 2, is the shape parameter. The third argument, 0.8, is the scale parameter in subjective time units. The value of 0.8 is chosen because it maximizes the model’s correspondence to the observed profiles. The *k* at the beginning of the expression for the inverted gamma distribution in Eq. 5 adjusts the vertical scale to the difference between the response rates during ~T and T intervals, that is, to the magnitude of the drop in response rate during the Tones. This magnitude depends strongly on the informativeness.

With the scale parameter set to 0.8, the model in Eq. 5 generates response-rate profiles very like the observed data; compare right and left columns Fig. 5. On this model, 55% of the drops in response rate occur within the first subjective moment after CS offset. When informativeness is 1.5 or less, that is, when the Tone occupies a relatively small part of the Tone–no Tone cycle, Tone offset occurs less than three-fourths of the way through the first subjective moment (Fig. 5, top). This means that Tone offset closes the window on the profile in Eq. 5 before the response rate begins to rise. On the other hand, when ι_~T_ = 9 (Fig. 5, bottom), the Tone occupies most of a Tone–no Tone cycle, so Tones last 1.74 subjective moments. In that case, the window of width *T* on the convolution in Eq. 5 includes much of the phase during which the recovery exponential dominates and the average response rate rises back toward the ~T rate (Fig. 5). Further explanation of this model, justifications for its assumptions, and code implementing it are in the Supplementary Materials.

### Mesolimbic dopamine encodes reward availability states

**Fig. 6.**
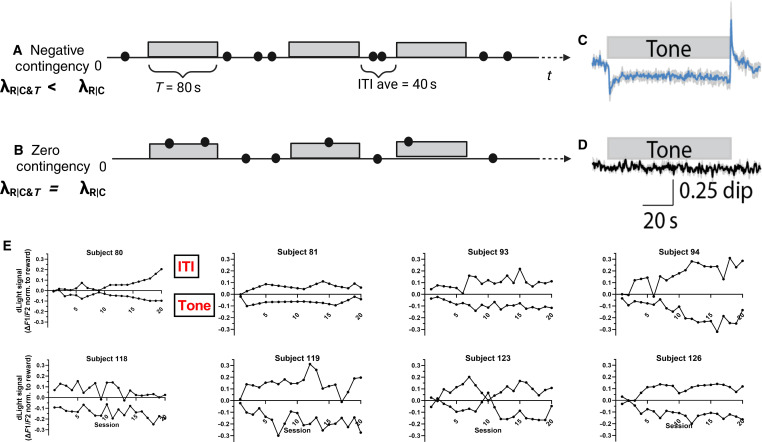
Dopamine release during negative contingency and zero contingency operant protocols. (**A**) Schematic of the operant negatively contingent paradigm in which mice can earn rewards for lever presses (black dots) made on an RI20 schedule during the absence of Tone, ITI average = 40 s. No rewards can be earned in the presence of the Tone, *T* = 80 s. Tone offset provides information about change in reward rate with an informativeness equal to the ratio of the reward rate in the absence of Tone (3/min) and the contextual reward rate over the entire session (1/min). ɩ = 3. (**B**) Schematic of a zero-contingency protocol used for a control group of mice. Rewards could be earned on an RI60 schedule throughout the session, and the presence of the 80-s Tone had no relevance (ɩ = 1). (**C**) Dopamine in the NAc, measured by dLight is lower during the 80-s Tone period compared to the ITI period in mice that have been trained on the negatively contingent protocol. (**D**) Dopamine in the NAc is not modulated by the Tone in the zero-contingency mice. (**E**) The average value for dLight during ITI and S- periods (normalized to dLight reward response within subject and session) for each daily session for eight mice trained in the negatively contingent task, demonstrating that dopamine is consistently lower during the Tone than during the ITI, and this difference emerges quickly.

The relationship between the informativeness of negatively contingent cues and learning is not restricted to Pavlovian conditioning. Cue informativeness also predicts the rate and depth of learning when responses are required to earn reinforcers during the ITI (*44*). In the context of such stimulus controlled instrumental responding, consistent with the idea that animals encode metric temporal structure and respond to the informativeness of cues that predict changes in reward rates, the activity of mesoaccumbal dopamine cells, and dopamine release in the nucleus accumbens (NAc) reflects reward availability state (*50*). Mice were trained in an operant conditioning paradigm with the same negative contingency as the classical conditioning paradigm depicted in Fig. 1D. Specifically, mice could earn rewards on a variable interval 20-s schedule except when a Tone of 80-s duration was presented. During the S^−^ Tone, no rewards could be earned (Fig. 6A). Extracellular dopamine level in the ventral striatum (NAc) was monitored during the task via fiber photometric recordings of fluorescence from the genetically encoded dopamine biosensor, dLight (*51*). Consistent with our information theoretic explanation of learning, the critically informative events (Tone offset and Tone onsets) were accompanied by large rapid transients in dopamine that represent transitions between reward availability states. Negative transients occurred when Tone onset signaled the transition to no reward availability and positive transients occurred when Tone offset signaled the transition to reward availability (Fig. 6B). No Tone-related fluctuations in dopamine occurred in mice that had been trained in a zero-contingency protocol (Fig. 6, C and D). In addition to the rapid dopamine transients at Tone onset and offset in conditioned mice, tonic dopamine levels were also influenced by reward availability states. Dopamine level during the Tone was lower than during the ITI (Fig. 6B). We have previously shown that the difference in dopamine level during the S^−^ Tone and the ITI cannot be accounted for by movement-related striatal dopamine release (*50*). Dopamine Tone quickly reflected reward availability states typically after only one or two training sessions (Fig. 6E). Once separated, the Tone and ITI dopamine states were consistently symmetrically opposed across 20 training sessions.

## DISCUSSION

Here, we show that the metric temporal structure of how cues relate to rewards determines the rate of learning and predicts behavior quantitatively. This temporal information is used to compute an objective parameter that we refer to as informativeness (*43*), the ratio of the contextual rate to the higher rate of reinforcement that occurs during the CS in positive-contingency protocols and during the ~CS intervals in negative-contingency protocols. We term this parameter informativeness because its logarithm is the mutual information, the number of bits communicated by the CS. When there are no background reinforcements in a one-CS protocol, the informativeness is the *C/T* ratio, the ratio of the interval between reinforcements in the context (C) and the expected interval when the CS is also present. When this ratio is very large, conditioning occurs in a single trial (*52*).

The original data from Gibbon and Balsam (*47*) and Jenkins *et al.* (*52*) came from pigeon autoshaping experiments, but the effect of *C/T* on learning is a fundamental quantitative and universal phenomenon. It has been demonstrated in rabbit eyeblink conditioning [(*48*); see figure 10], head-fixed mice (*49*), and rat inhibitory conditioning (this paper).

**Fig. 7.**
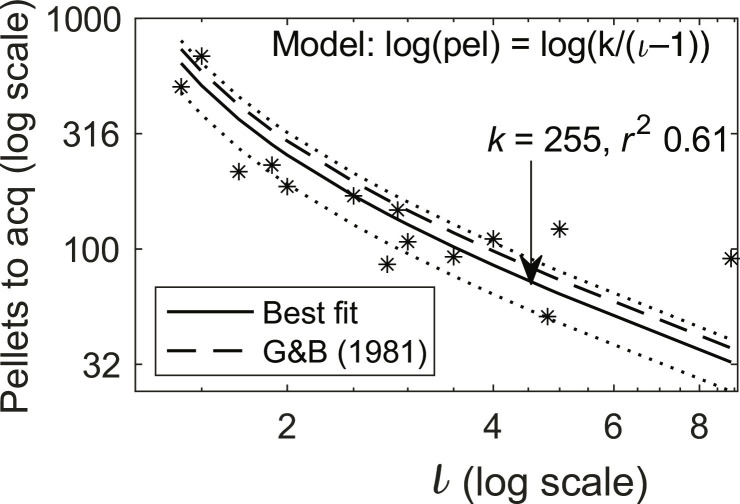
Acquisition as a function of temporal informativeness. Rat inhibition and pigeon excitation data fit the same model. The solid line is the best-fit regression, and the dotted lines are the confidence limits for the rat inhibitory conditioning data presented in this study. The dashed line is the regression from pigeon excitatory autoshaping presented by Gibbon and Balsam (*47*) (scale = log-log). (Acquisition is defined as the cumulative number of pellets delivered when the strength of the evidence for a negative difference in Tone poke rate - ITI poke rate had significance α = 0.01.)

Our quantitative law with a single, well-established parameter of learning fits behavioral data across species, protocols, and types of reinforcement with a level of precision that is unprecedented in the 100-year history of the study of associative learning. This is illustrated in Fig. 7, which shows that data from pigeon excitatory autoshaping fits within the confidence limits of the best-fit regression for the rat inhibitory conditioning presented in this study.

It is also worth noting that in negative-contingency protocols, cues that are never reinforced are typically referred to as “inhibitory,” but we show that such cues do not typically inhibit behavior. Average response rates during Tones do not decrease with training; rather, training increases responding during the ITI’s in response to the information provided by Tone offset. Responding during the Tones either remains the same or increases in some subjects even after many unreinforced Tones. In a minimally informative (ɩ = 1.5) negative-contingency protocol (#9 in Table 1), after 4000 pellet deliveries during the ITIs and no pellet deliveries during as many Tones, rates of responding during the Tones and ITIs were both well above their initial values at the start of training for most subjects. At asymptote, response rates were only slightly lower during the Tones compared to the ITIs. Any trial- or state-based model of associative learning in which nonreinforcements are regarded as “NoUS” events that decrement associative strength or state value cannot explain why the 4000 (unobservable) NoUSs that hypothetically occurred at unspecifiable times during the 4000 unreinforced Tones increased rate of responding during the Tones to a rate only slightly lower than the increased rate during the ITIs, which were observably reinforced 4000 times.

By comparing protocols in which the temporal metrics varied, we show that the informativeness of a protocol, not the absolute delay of reinforcement nor its probability, is the principal determinant of the difference between the average response rates during Tones and ITIs and of rate of learning. Our results are inconsistent with the assumption that delay reduces reinforcement efficacy and with the assumption that probability of reinforcement increases rate of learning when measured by trials to acquisition. When informativeness was low, learning was often extremely slow taking many hundreds of trials even though one pellet was delivered per ITI (on average), and no pellets were ever delivered during Tones.

In excitatory conditioning—positively contingent protocols in which every T is reinforced—when trials (hence reinforcements) to acquisition are plotted against informativeness on double logarithmic coordinates, a linear regression with a slope of −1 describes the data [(*48*), figure 9]. The slope agreement between that regression and the one we now report (Fig. 4A) reinforces the conclusion that inhibitory conditioning is excitatory conditioning with the roles of Tone onset and offset reversed. Increasing informativeness 10-fold increases rate of learning 10-fold in both cases, without changing the probabilities of reinforcement and nonreinforcement. This finding presents a formidable challenge to trial- and state-based models of associative learning, models in which learning is driven by the probability of reinforcement and nonreinforcement.

### Learning is timescale invariant

In our negatively contingent protocols, learning is timescale invariant. For example, subjects show similar learning rates in protocols with the same informativeness (ɩ = 2), even when the Tone durations differ by a factor of 4 (protocols T20:ITI20 and T80:ITI80 in Fig. 3B). By contrast, a protocol with a Tone of the same duration (80 s), but is more informative (ɩ = 5), produces faster learning. Similarly, the results from four protocols with two different ITI durations, each with two different Tone durations (Fig. 3D), reveal that varying informativeness has the same effect on learning rate whether done by varying *T* or ITI (and thereby μ_*R*∣C&~T_). While our results demonstrate timescale-invariant learning in Pavlovian inhibitory protocols, timescale invariance of learning was first shown for excitatory conditioning in pigeons [(*47*) and see figure 4 in (*45*)].

One interesting implication of the scalar dependency of rate of learning on informativeness is that when informativeness is substantially greater than 100, an appetitive conditioned response to a predictive event appears after the subject’s first experience of the event’s predictive power (*52*)—one trial learning for food reinforcement. Thus, one must consider the differences in informativeness of the protocols typically used with appetitive versus aversive reinforcers before concluding that appetitive and aversive reinforcers (e.g., food, water, poison, and shock) produce different rates of learning.

In addition to our behavioral results challenging trial- and state-based models of learning, we show that such models do not explain conditioning-related dopamine release. Temporal pairing–based reinforcement models predict that because of the discrete imaginary NoUS trials within the S^−^, dopamine level should increase in magnitude across the cue because with each nonrewarded segment of time, the prediction of reward (value state) should increase (*53*). Our continuous monitoring of extracellular dopamine revealed no such predictive “ramping.” Our result is consistent with the hypothesis that animals encode the rate of reward availability within the temporal structure of the protocol.

The findings we present and reference here require a broad scale rethinking of how learning is instantiated by the brain. Our work shows that the neurobiological mechanisms of associative learning must involve learning and using the metric temporal structure of events: Subjects measure temporal intervals that differ by many orders of magnitude; they store the results of their measurements in memory; and they perform arithmetic operations (≥, ×, ÷) on the remembered intervals when generating appropriate anticipatory behavior. A model based on these behaviorally derived insights, such as Eq. 2, provides a parameter-free analytic explanation of credit assignment (*27*), without invoking nonevents occurring at unspecifiable times. Informativeness plays a fundamental role in it because the temporal information communicated by a predictive event is the log of its informativeness (Eq. 1). Informativeness is a unitless ratio, which explains why associative learning is timescale invariant (*48*).

Other, recent work includes parameters other than pairing in explanatory models of learning (*21*, *49*, *54*). Biological mechanisms capable of the necessary computations are yet to be found. Some suggested mechanisms are based on the idea that information may be encoded by cell-intrinsic properties with RNA, DNA, histones, or kinases as substrates (*55*–*60*). We do not yet know whether any of these mechanisms are suitable for encoding the metric structure of experiences.

Here, we focused on learning the metric temporal structure of experience but other sorts of information are contained in associative memories, some quantitative (e.g., spatial location, reinforcement value, and sensory intensity) and some categorical (e.g., type of reinforcement). The mechanisms of storage of any of this specific information have not yet been identified. Regardless of the biological nature of learning, the broader the inclusion of behavioral work on associative learning, the more likely a universal mechanism will be found. In contrast, focusing on only select behavioral observations will impede progress in understanding of brain-behavior mechanisms. Given the overwhelming evidence that learning depends on encoding metric temporal structure, a focus on identifying neural representations of time may be the most constructive approach.

## MATERIALS AND METHODS

### Pavlovian conditioning

Animal work was approved by the Institutional Animal Care and Use committees at Columbia University Medical Center (IACUC protocol number AAAM9903) and the New York State Psychiatric Institute (IACUC protocol 1679).

Male, Sprague-Dawley rats were housed in groups of two in a colony room on a 12:12-hour light:dark cycle. Water was available ad lib in the home cages. They were fed in their home cages for 1 hour after experimental sessions, which occurred 5 days per week. On weekends, they had ad lib access to food until approximately 22 hours before the first weekday session. They were approximately 2 months old at the start of training and had been handled for 1 week before that. They were trained in eight identical experimental chambers (30.5 cm by 24.1 cm by 21.0 cm) located in ventilated and soundproof boxes. Each chamber was equipped with a speaker, a house light, and a pellet dispenser (Model ENV-203, Med Associates), which delivered 20-mg pellets into a head entry–detecting trough (Models ENV-200R7 and ENV-254-CB, Med Associates). They initially received two sessions of magazine training, during which 40 pellets were delivered at random times during a 20-min session (random-time 30-s schedule), followed by daily sessions with one of the experimental protocols. The data were collected in six different experiments. Rats were randomly assigned to experimental groups within each experiment. Table 1 gives the parameters for the 26 protocols (session durations, trials/session, etc) used in the experiments. The Tones were 1000 Hz at 80 decibels. Tones were of fixed duration except in experiment 5 in which two of the groups were exposed to variable duration Tones. In these two groups, durations were sampled from an exponential distribution with the means indicated in Table 1. ITIs and food delivery times were always of variable durations drawn from exponential distributions. The time of occurrence of each head entry and the time of onset and termination of all stimulus events were recorded with 0.1-s resolution. All head entries within 2 s following a pellet delivery were excluded from the analyses so the measures reflected anticipatory responding and not pellet-evoked responding. All dependent variables, raw data, and data-processing code have been uploaded to Dryad: https://doi.org/10.5061/dryad.3xsj3txq8.

### Operant conditioning with dopamine recording

The methods for this experiment have previously been described in detail in (*50*). Briefly, Male C57BL/6 J wild-type mice were injected with AAV5-CAG-dLight1.1 (200 nl; Addgene) in the NAc and implanted with 400-μm optical fibers (Doric). Animals recovered for 2 to 3 weeks before behavioral testing, and recordings began at least 4 weeks after surgery.

Behavior apparatus: Experimental chambers had a single lever to the left of a feeder trough centered on one side of the chamber (Med Associates). Speakers delivered the S^−^ at 3.5 kHz and 80 dB and were positioned on the wall opposite the lever and the food port.

Behavior paradigm: Animals were pretrained to press a lever initially on a continuous reward schedule and then on an RI schedule of an average of 5, 10, and lastly 20 s between each reward [as described previously (*44*)]. Animals were rewarded with 20 μl of evaporated milk. Tone training commenced after animals consistently earned 40 rewards on a RI20 schedule in less than 30 min. For the conditioned group, a training session included 20× 80-s-long presentations of the Tone with an average ITI of 40 s (range 1.5 to 90 s, drawn from an exponential-like distribution). In between Tone presentations (during the ITI), rewards could be earned on an RI 20-s schedule. For the random group, training sessions similarly included 20× 80-s-long presentations of a Tone. Rewards could be earned at any time during the session, including during the Tone, on an RI60 schedule, which was chosen so that both groups of animals could earn similar numbers of rewards across each session.

Dopamine recordings: In vivo fluorescence was continuously monitored throughout the behavioral sessions using optical components (Doric Lenses) controlled by an RZ5P acquisition processor (TDT). Two photodiodes (405 and 465 nm) were sinusoidally pulsed (at 210 and 330 Hz, respectively; if two animals were recorded from simultaneously, photodiodes for animal 2 were pulsed at 270 and 450 Hz, respectively). Traces were demodulated online. The times of behavioral variables were recorded as TTL inputs to the acquisition system and through the MED-PC system. Data were analyzed using custom MATLAB scripts. To correct for potential motion artifact or within session photobleaching, we normalized the dLight-specific signal generated by 465-nm excitation to the isosbestic signal generated by 405-nm excitation (*61*). Specifically, we calculated Δ*F*1/*F*2 by taking a least squares linear fit of the 405-nm channel value aligned to the 465-nm channel. Signals were background subtracted using a 0.5-s window immediately before each event. To normalize for signal recovery for each mouse on each experimental day, DA responses to the S− were normalized to the average peak response to reward for each day of recording.

#### Statistics

The strength of the evidence for a negative slope in the cumulative record of the difference in estimated poke rates, $\sum_{i=1}^{i=n}\left({\hat{\lambda }}_{r|C\&T}-{\hat{\lambda }}_{r|C\&∼T}\right)$ , was computed after each pellet delivery, using the cumulative coding cost statistic: $nD_{\text{KL}}\left({\hat{\lambda }}_{r|C\&T}\|{\hat{\lambda }}_{r|C\&∼T}\right)$ , where *n* is the cumulative number of responses made during Tones and $D_{\text{KL}}\left({\hat{\lambda }}_{r|C\&\text{CS}}\|{\hat{\lambda }}_{r|C\&∼T}\right)$ is the Kullback-Leibler divergence of an exponential distribution with parameter ${\hat{\lambda }}_{r|C\&T}$ from one with parameter ${\hat{\lambda }}_{r|C\&∼T}$ . $nD_{\text{KL}}\left({\hat{\lambda }}_{r|C\&T}\|{\hat{\lambda }}_{r|C\&∼T}\right)\;\text{is}∼\Gamma \left(.5,1\right)$ under the null hypothesis. The Bayesian estimates of the rate parameters used the Jeffreys prior. More information about acquisition rates calculated using this method can be obtained from the data tables available at https://doi.org/10.5061/dryad.3xsj3txq8 including five different acquisition criteria for USs and trials to acquisition: the maximum of the cumulative difference function (which estimates onset of acquisition) and four increasingly stringent criteria on the strength of the evidence that acquisition has occurred (*P* < 0.1, *P* < 0.05, *P* < 0.01, and *P* < 0.001).

## References

[1] R. A. Rescorla, A. R. Wagner, in *Classical Conditioning II*, A. H. Black, W. F. Prokasy, Eds. (Appleton-Century-Crofts, 1972), pp. 64–99.

[2] R. S. Sutton, A. G. Barto, Toward a modern theory of adaptive networks: Expectation and prediction. Psychol. Rev. 88, 135–170 (1981). 7291377

[3] R. C. Honey, D. M. Dwyer, A. F. Iliescu, HeiDI: A model for Pavlovian learning and performance with reciprocal associations. Psychol. Rev. 127, 829–852 (2020). 32271046 10.1037/rev0000196

[4] E. H. Vogel, F. P. Ponce, A. R. Wagner, The development and present status of the SOP model of associative learning. Q. J. Exp. Psychol. 72, 346–374 (2019). 10.1177/1747021818777074 29741452

[5] P. Dayan, Y. Niv, Reinforcement learning: The good, the bad and the ugly. Curr. Opin. Neurobiol. 18, 185–196 (2008). 18708140 10.1016/j.conb.2008.08.003

[6] V. François-Lavet, P. Henderson, R. Islam, M. G. Bellemare, J. Pineau, An introduction to deep reinforcement learning. FNT Mach. Learn. 11, 219–354 (2018).

[7] I. Momennejad, E. M. Russek, J. H. Cheong, M. M. Botvinick, N. D. Daw, S. J. Gershman, The successor representation in human reinforcement learning. Nat. Hum. Behav. 1, 680–692 (2017). 31024137 10.1038/s41562-017-0180-8 PMC6941356

[8] M. G. Mattar, N. D. Daw, Prioritized memory access explains planning and hippocampal replay. Nat. Neurosci. 21, 1609–1617 (2018). 30349103 10.1038/s41593-018-0232-z PMC6203620

[9] N. Rouhani, Y. Niv, Signed and unsigned reward prediction errors dynamically enhance learning and memory. eLife 10, e61077 (2021). 33661094 10.7554/eLife.61077 PMC8041467

[10] A. R. Delamater, S. Oakeshott, Learning about multiple attributes of reward in Pavlovian conditioning. Ann. N. Y. Acad. Sci. 1104, 1–20 (2007). 17344542 10.1196/annals.1390.008

[11] J. A. Harris, D. W. S. Kwok, D. A. Gottlieb, The partial reinforcement extinction effect depends on learning about nonreinforced trials rather than reinforcement rate. J. Exp. Psychol. Anim. Learn. Cogn. 45, 485–501 (2019). 31368769 10.1037/xan0000220

[12] M. Haselgrove, A. Aydin, J. M. Pearce, A partial reinforcement extinction effect despite equal rates of reinforcement during Pavlovian conditioning. J. Exp. Psychol. Anim. Behav. Process. 30, 240–250 (2004). 15279514 10.1037/0097-7403.30.3.240

[13] M. E. Bouton, A. M. Woods, T. P. Todd, Separation of time-based and trial-based accounts of the partial reinforcement extinction effect. Behav. Processes 101, 23–31 (2014). 23962669 10.1016/j.beproc.2013.08.006 PMC3926907

[14] P. D. Balsam, C. R. Gallistel, Temporal maps and informativeness in associative learning. Trends Neurosci. 32, 73–78 (2009). 19136158 10.1016/j.tins.2008.10.004 PMC2727677

[15] C. R. Gallistel, Conditioning from an information processing perspective. Behav. Processes 62, 89–101 (2003). 12729971 10.1016/s0376-6357(03)00019-6

[16] J. T. Wilkes, C. R. Gallistel, in *Computational Models of Brain and Behavior*, A. A. Moustafa, Ed. (John Wiley & Sons, Ltd, 2017), pp. 481–492.

[17] P. D. Balsam, M. R. Drew, C. R. Gallistel, Time and associative learning. Comp. Cogn. Behav. Rev. 5, 1–22 (2010). 21359131 10.3819/ccbr.2010.50001 PMC3045055

[18] M. Molet, G. Miguez, H. X. Cham, R. R. Miller, When does integration of independently acquired temporal relationships take place? J. Exp. Psychol. Anim. Behav. Process. 38, 369–380 (2012). 22905828 10.1037/a0029379

[19] B. Crimmins, T. J. Burton, M. McNulty, V. Laurent, G. Hart, B. W. Balleine, Response-independent outcome presentations weaken the instrumental response-outcome association. J. Exp. Psychol. Anim. Learn. Cogn. 48, 396–412 (2022). 36265026 10.1037/xan0000340

[20] R. A. Rescorla, Unequal associative changes when excitors and neutral stimuli are conditioned in compound. Q. J. Exp. Psychol. B 54, 53–68 (2001). 11216301 10.1080/02724990042000038

[21] L. T. Coddington, S. E. Lindo, J. T. Dudman, Mesolimbic dopamine adapts the rate of learning from action. Nature 614, 294–302 (2023). 36653450 10.1038/s41586-022-05614-z PMC9908546

[22] R. A. Rescorla, Probability of shock in the presence and absence of CS in fear conditioning. J. Comp. Physiol. Psychol. 66, 1–5 (1968). 5672628 10.1037/h0025984

[23] R. A. Rescorla, J. C. Skucy, Effect of response-independent reinforcers during extinction. J. Comp. Physiol. Psychol. 67, 381–389 (1969).

[24] R. A. Rescorla, Pavlovian conditioning and its proper control procedures. Psychol. Rev. 74, 71–80 (1967). 5341445 10.1037/h0024109

[25] R. A. Rescorla, Conditioned Inhibition of fear resulting from negative CS-US contingencies. J. Comp. Physiol. Psychol. 67, 504–509 (1969). 5787403 10.1037/h0027313

[26] R. A. Rescorla, Pavlovian conditioning. It’s not what you think it is. Am. Psychol. 43, 151–160 (1988). 3364852 10.1037//0003-066x.43.3.151

[27] C. R. Gallistel, *The Organization of Learning* (The MIT Press, 1990); psycnet.apa.org.

[28] C. R. Gallistel, L. D. Matzel, The neuroscience of learning: Beyond the Hebbian synapse. Annu. Rev. Psychol. 64, 169–200 (2013). 22804775 10.1146/annurev-psych-113011-143807

[29] C. R. Gallistel, P. D. Balsam, Time to rethink the neural mechanisms of learning and memory. Neurobiol. Learn. Mem. 108, 136–144 (2014). 24309167 10.1016/j.nlm.2013.11.019 PMC3932565

[30] J. Gibbon, L. Farrell, C. M. Locurto, H. J. Duncan, H. S. Terrace, Partial reinforcement in autoshaping with pigeons. Anim. Learn. Behav. 8, 45–59 (1980).

[31] W. O. Jenkins, J. C. Stanley, Partial reinforcement: A review and critique. Psychol. Bull. 47, 193–234 (1950). 15417676 10.1037/h0060772

[32] D. J. Lewis, Partial reinforcement: A selective review of the literature since 1950. Psychol. Bull. 57, 1–28 (1960). 14416507 10.1037/h0040963

[33] I. Gormezano, S. R. Coleman, Effects of partial reinforcement on conditioning, conditional probabilities, asymptotic performance, and extinction of the rabbit’s nictitating membrane response. Pavlov. J. Biol. Sci. 10, 13–22 (1975). 1114026 10.1007/BF03000620

[34] C. K. J. Chan, J. A. Harris, The partial reinforcement extinction effect: The proportion of trials reinforced during conditioning predicts the number of trials to extinction. J. Exp. Psychol. Anim. Learn. Cogn. 45, 43–58 (2019). 30604994 10.1037/xan0000190

[35] D. A. Gottlieb, Acquisition with partial and continuous reinforcement in pigeon autoshaping. Learn. Behav. 32, 321–334 (2004). 15672827 10.3758/bf03196031

[36] D. A. Gottlieb, Is the number of trials a primary determinant of conditioned responding? J. Exp. Psychol. Anim. Behav. Process. 34, 185–201 (2008). 18426303 10.1037/0097-7403.34.2.185

[37] P. Balsam, Relative time in trace conditioning. Ann. N. Y. Acad. Sci. 423, 211–227 (1984). 6588787 10.1111/j.1749-6632.1984.tb23432.x

[38] G. E. Schafe, S. I. Sollars, I. L. Bernstein, The CS–US interval and taste aversion learning: A brief look. Behav. Neurosci. 109, 799–802 (1995). 7576224

[39] J. D. Raybuck, K. M. Lattal, Bridging the interval: Theory and neurobiology of trace conditioning. Behav. Processes 101, 103–111 (2014). 24036411 10.1016/j.beproc.2013.08.016 PMC3943893

[40] W. H. Meck, R. M. Church, Discrimination of intertrial intervals in cross-modal transfer of duration. Bull. Psychon. Soc. 19, 234–236 (1982).

[41] E. A. Franz, H. N. Zelaznik, A. Smith, Evidence of common timing processes in the control of manual, orofacial, and speech movements. J. Mot. Behav. 24, 281–287 (1992). 12736133 10.1080/00222895.1992.9941623

[42] C. R. Gallistel, Robert Rescorla: Time, information and contingency. Revista de historia de la psicología. 42, 7–21 (2021).

[43] P. D. Balsam, S. Fairhurst, C. R. Gallistel, Pavlovian contingencies and temporal information. J. Exp. Psychol. Anim. Behav. Process. 32, 284–294 (2006). 16834495 10.1037/0097-7403.32.3.284

[44] A. Kalmbach, E. Chun, K. Taylor, C. R. Gallistel, P. D. Balsam, Time-scale-invariant information-theoretic contingencies in discrimination learning. J. Exp. Psychol. Anim. Learn. Cogn. 45, 280–289 (2019). 31021132 10.1037/xan0000205 PMC7771212

[45] C. R. Gallistel, P. E. Latham, Bringing Bayes and Shannon to the study of behavioural and neurobiological timing and associative learning. Timing Time Percept. 11, 29–89 (2022).

[46] C. R. Gallistel, A. R. Craig, T. A. Shahan, Contingency, contiguity, and causality in conditioning: Applying information theory and Weber’s Law to the assignment of credit problem. Psychol. Rev. 126, 761–773 (2019). 31464474 10.1037/rev0000163

[47] J. Gibbon, P. Balsam, “Spreading association in time” in *Autoshaping and Conditioning Theory*, C. M. Locurto, H. S. Terrace, J. Gibbon, Eds. (Academic Press, 1981), pp. 219–253.

[48] C. R. Gallistel, J. Gibbon, Time, rate, and conditioning. Psychol. Rev. 107, 289–344 (2000). 10789198 10.1037/0033-295x.107.2.289

[49] H. Jeong, A. Taylor, J. R. Floeder, M. Lohmann, S. Mihalas, B. Wu, M. Zhou, D. A. Burke, V. M. K. Namboodiri, Mesolimbic dopamine release conveys causal associations. Science 378, eabq6740 (2022). 36480599 10.1126/science.abq6740 PMC9910357

[50] A. Kalmbach, V. Winiger, N. Jeong, A. Asok, C. R. Gallistel, P. D. Balsam, E. H. Simpson, Dopamine encodes real-time reward availability and transitions between reward availability states on different timescales. Nat. Commun. 13, 3805 (2022). 35778414 10.1038/s41467-022-31377-2 PMC9249893

[51] T. Patriarchi, J. R. Cho, K. Merten, M. W. Howe, A. Marley, W.-H. Xiong, R. W. Folk, G. J. Broussard, R. Liang, M. J. Jang, H. Zhong, D. Dombeck, M. von Zastrow, A. Nimmerjahn, V. Gradinaru, J. T. Williams, L. Tian, Ultrafast neuronal imaging of dopamine dynamics with designed genetically encoded sensors. Science 360, eaat4422 (2018). 29853555 10.1126/science.aat4422 PMC6287765

[52] H. M. Jenkins, R. A. Barnes, F. J. Barrera, in *Autoshaping and Conditioning Theory*, C. M. Locurto, H. S. Terrace, J. Gibbon, Eds. (Academic Press, 1981), pp. 255–284.

[53] H. R. Kim, A. N. Malik, J. G. Mikhael, P. Bech, I. Tsutsui-Kimura, F. Sun, Y. Zhang, Y. Li, M. Watabe-Uchida, S. J. Gershman, N. Uchida, A unified framework for dopamine signals across timescales. Cell 183, 1600–1616.e25 (2020). 33248024 10.1016/j.cell.2020.11.013 PMC7736562

[54] L. Qian, M. Burrell, J. A. Hennig, S. Matias, V. N. Murthy, S. J. Gershman, N. Uchida, The role of prospective contingency in the control of behavior and dopamine signals during associative learning. bioRxiv 2024.02.05.578961 [**Preprint**]. [6 February 2024]. 10.1101/2024.02.05.578961. PMC12148708 40102680

[55] R. S. Moore, R. Kaletsky, C. Lesnik, V. Cota, E. Blackman, L. R. Parsons, Z. Gitai, C. T. Murphy, The role of the *Cer1* transposon in horizontal transfer of transgenerational memory. Cell 184, 4697–4712.e18 (2021). 34363756 10.1016/j.cell.2021.07.022 PMC8812995

[56] H. Akhlaghpour, An RNA-based theory of natural universal computation. J. Theor. Biol. 537, 110984 (2022). 34979104 10.1016/j.jtbi.2021.110984

[57] C. A. Miller, C. F. Gavin, J. A. White, R. R. Parrish, A. Honasoge, C. R. Yancey, I. M. Rivera, M. D. Rubio, G. Rumbaugh, J. D. Sweatt, Cortical DNA methylation maintains remote memory. Nat. Neurosci. 13, 664–666 (2010). 20495557 10.1038/nn.2560 PMC3043549

[58] J. J. Day, J. D. Sweatt, DNA methylation and memory formation. Nat. Neurosci. 13, 1319–1323 (2010). 20975755 10.1038/nn.2666 PMC3130618

[59] S. Peleg, F. Sananbenesi, A. Zovoilis, S. Burkhardt, S. Bahari-Javan, R. C. Agis-Balboa, P. Cota, J. L. Wittnam, A. Gogol-Doering, L. Opitz, G. Salinas-Riester, M. Dettenhofer, H. Kang, L. Farinelli, W. Chen, A. Fischer, Altered histone acetylation is associated with age-dependent memory impairment in mice. Science 328, 753–756 (2010). 20448184 10.1126/science.1186088

[60] C. Adaikkan, K. Rosenblum, A molecular mechanism underlying gustatory memory trace for an association in the insular cortex. eLife 4, e07582 (2015). 26452094 10.7554/eLife.07582 PMC4703067

[61] T. N. Lerner, C. Shilyansky, T. J. Davidson, K. E. Evans, K. T. Beier, K. A. Zalocusky, A. K. Crow, R. C. Malenka, L. Luo, R. Tomer, K. Deisseroth, Intact-brain analyses reveal distinct information carried by SNc dopamine subcircuits. Cell 162, 635–647 (2015). 26232229 10.1016/j.cell.2015.07.014 PMC4790813

