# Supplementary material for: Learning depends on the information conveyed by temporal relationships between events and is reflected in the dopamine response to cues
Source: Sci Adv. 2024 Sep 6;10(36):eadi7137. doi: 10.1126/sciadv.adi7137 (PMC11378905; doi:10.1126/sciadv.adi7137)
Supplement: Supplementary file 1 — Supplementary Text Figs. S1 to S3 [file sciadv.adi7137_sm.pdf]

Science Advances
AAAS

Supplementary Materials for

Learning depends on the information conveyed by temporal relationships between events and is reflected in the dopamine response to cues

Peter D. Balsam *et al.*

Corresponding author: Peter D. Balsam, pbalsam@barnard.edu

Sci. Adv. **10**, eadi7137 (2024)
DOI: 10.1126/sciadv.adi7137

This PDF file includes:

Supplementary Text
Figs. S1 to S3

Supplemental Material

Model of pattern of responding during the CS

In Negatively contingent protocols, higher response rates during the ITIs drop to lower rates at varying latencies following CS onsets. We assume the distribution of these latencies to be exponential (Figure S1a, red curve)—with a time constant, μ, that is proportional to the duration, C, of the average cycle. In the protocols of the current experiments, the cycle is the total time that it takes from the start of one CS to the start of the next one ($D_{CS} + D_{ITI}$). When CS termination time is fixed, it can be anticipated, subjects resume the higher rate of responding before the termination of the CS, but only on some trials. We assume the resumption latencies to also be exponential and with the same time constant as the drop latencies (green curves in Figure S1 a & b). Then, the convolution of the exponential distribution with itself yields the probability of responding at the lower rate as a function of elapsed time in the CS (Figure S1c). The convolution distribution is the gamma distribution with shape parameter 2 and scale parameter 2μ.

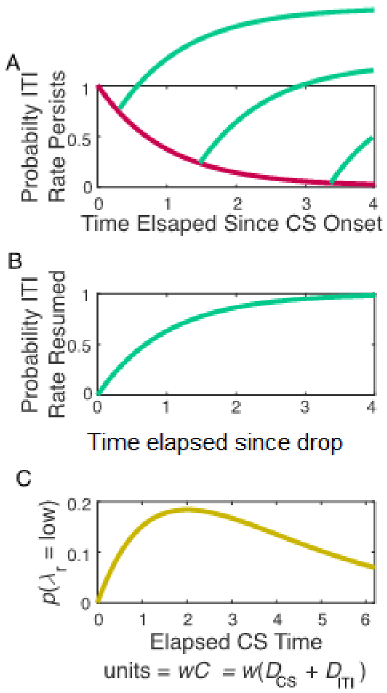

Figure S1. Within-CS response pattern. A. *The drop latencies after CS onset are exponentially distributed with a time constant, μ, proportionate to the cycle duration (red distribution).* B. *The resumption latencies from the time of the drop are also exponentially distributed with the same time constant (green cumulative distribution, shown here and in a)* C. *The probability that the subject is responding at the lower rate at a given moment in time. The units on the abscissa are the subjective time units. The Weber fraction for duration, w, determines the relation between subjective time units and time measured in seconds (see text for more explanation).*

The properly scaled vertical flip of the yellow gamma distribution in Figure 1c gives the shape of the within-CS response profile when CS duration is 4 times longer than average ITI duration. When CS duration is short relative to average ITI duration, the duration of the CS is short in relation to the time scale of the convolution; hence, a smaller portion of the convolution falls within the CS (Figure S2, dashed and dot-dashed curves, and Figure S3 A&C). When CS duration is long relative to ITI duration, more of the convolution falls within the CS (Figure S2, solid curve and Figure S3B).

Figure S2. Convolution of drop and resumption latency distributions. *The gamma(2,2μ) distribution is cropped from the right by CS termination (heavy vertical line). The extent of the cropping is jointly determined by μ and CS duration relative to the cycle duration. When the CS is only 2μ long, the profile looks like that in Figure S3 A&C; when it is 6μ long, it looks like that in Figure S3 B.*

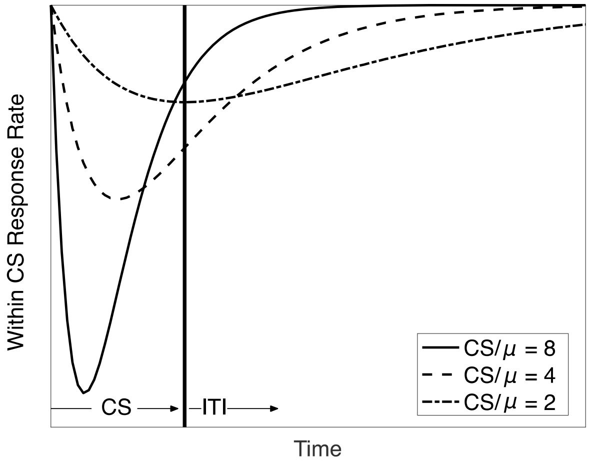

Figure S3. Model fits to data. Empirically *observed profiles are shown by dotted curves with solid (vertical) standard error bars. The model's ability to capture the shape of the observed profile is shown by the dashed curves. In all 3 plots, the value assumed for the Weber fraction, w, is 0.125 and μ = wC, where C is the average duration of the duty cycle.* A. *C = 40s and CS duration is 20s.* B. *C = 100s and CS duration is 80s.* C. *C = 160s and CS duration is 80s. The fact that the observed shape in Panel C is similar to that in Panel A and not to that in Panel B, even though CS duration is 80s in both the B and C panels, is the time-scale invariance of the profiles.*

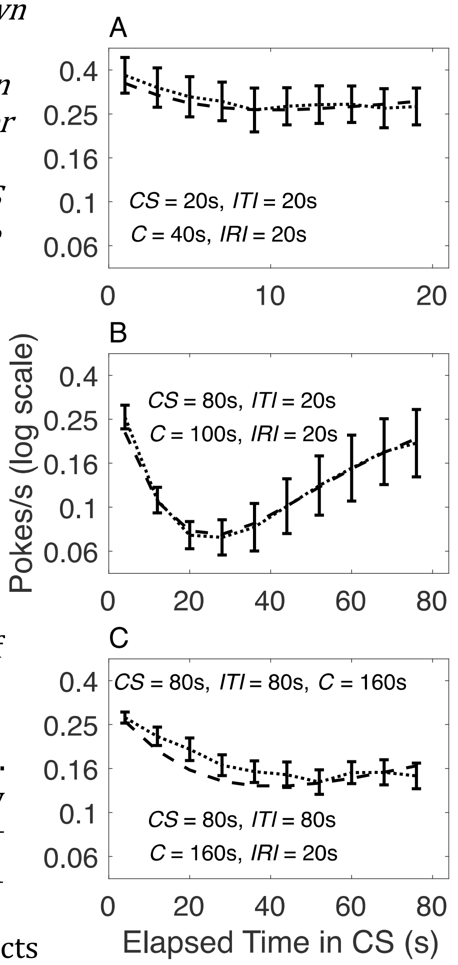

The Weber fraction is the fraction of a reference duration by which another duration must be increased or decreased to make it just noticeably longer or shorter. When *C* is the reference duration, then *wC* is one just noticeable difference (jnd). When *C* is measured in seconds, one subjective time unit lasts *wC* seconds. Empirically determined values of *w for time* in a variety of subject species mostly fall between 0.125 and 0.25 (Aydoğan et al., 2023). In the implementation of the model in Figure S3, we assumed the subjective time constant equals 1*μ* and *w* equals 0.125. Thus, from a subject-centered perspective, subjects respond quickly to CS onset regardless of the duration of the duty cycle because $1–e^{-1}$ = 63% of their response latencies are ≤ 1 subjective time unit (≤1 jnd). When *w* is taken to be greater than 0.125, then the value for *μ* that captures the profile shapes is less than 1 jnd. In that case, subjects respond even more quickly on their subjective time scale; 63% of the behavioral adjustments fall within a fraction of one subjective time unit.

Our model of the time-scale invariant decision latencies during the CS accords with the well-established time-scale invariance of the decision to begin responding in "excitatory" protocols with fixed CS duration and to stop responding in peak-procedure protocols (35, 55-58).

The counter-intuitive aspect of our model is that the timing of the latency to resume the ITI rate of responding in anticipation of CS termination starts as soon as a reduction in the rate of responding occurs. It is not anchored to the anticipated time at which the CS will end, as one might intuitively expect.

On this model, *resumptio*n latencies on trials with short drop latencies sometimes occur sooner (when measured from CS onset) than the *reductio*n latencies on trials with long drop latencies—no matter how long the fixed duration of the CS. When CS duration is very much longer than ITI duration, the decision to resume may become tethered to the anticipated time of CS termination. In the simple model that well describes the data in hand, that dies not occur. Indeed, as the ratio between the CS duration and the duration of the duty cycle approaches 1, the probability that the subject will have resumed more rapid responding by the end of the CS asymptotes at 0.81. This prediction of the model is readily tested by further parametric experiment.

Whatever the outcome of future experiments testing this model, we have found that, when measured in conventional units of time like the second, the longer the average duty cycle, the more slowly subjects make (or implement) both the decision to reduce their response rate during the CS intervals (intervals when no reinforcements occur) and the decision to increase it again when the time at which the CS terminates is predictable. We have also found, from experiments in which the CS duration was exponentially distributed, that when the time of termination cannot be anticipated, the latency to reduce the response rate following CS onset is exponentially distributed and resumptions do not occur (Variable CS groups in Table 1 of main text- protocols 11 and 13). Any hypothesized neurobiological basis of conditioned responding in "inhibitory" Pavlovian protocols must explain the strong dependence of decision latencies during the CS and the magnitude of the behavioral adjustment to it on the duration of the duty cycle, which may be orders of magnitude longer than the duration of the CS.
